# An Assessment of the Impact of Fortification of Staples and Condiments on Micronutrient Intake in Young Vietnamese Children

**DOI:** 10.3390/nu4091151

**Published:** 2012-08-24

**Authors:** Arnaud Laillou, Le Bach Mai, Le Thi Hop, Nguyen Cong Khan, Dora Panagides, Frank Wieringa, Jacques Berger, Regina Moench-Pfanner

**Affiliations:** 1 Global Alliance for Improved Nutrition (GAIN), rue de vermont 37-39, Geneva 1201, Switzerland; Email: dpanagides@gainhealth.org (D.P.); rmoenchpfanner@gainhealth.org (R.M.-P.); 2 National Institute of Nutrition (NIN), 48b Tang Bat Ho, Hanoi 10000, Vietnam; Email: bachmai_nin@yahoo.com (L.B.M.); lethihop@viendinhduong.vn (L.T.H.); 3 Vietnam Food Administration (VFA), Hanoi 10000, Vietnam; Email: dr_nguyen_cong_khan@yahoo.com; 4 UMR 204 “Prevention of Malnutrition and Associated Diseases”, IRD-UM2-UM1, Institute of Research for Development (IRD), BP 645, Montpellier cedex 34394, France; Email: franck.wieringa@ird.fr (F.W.); jacques.berger@ird.fr (J.B.)

**Keywords:** fortification, strategy, infant, young children, Vietnam, recommended dietary allowance

## Abstract

Targeted fortification programs for infants and young children are an effective strategy to prevent micronutrient deficiencies in developing countries, but the role of large-scale fortification of staple foods and condiments is less clear. Dietary modeling in children aged 6–60 months was undertaken, based on food consumption patterns described in the 2009 national food consumption survey, using a 24-h recall method. Consumption data showed that the median intake of a child for iron, vitamin A and zinc, as a proportion of the Vietnamese Recommended Dietary Allowance (VRDA), is respectively 16%–48%, 14%–49% and 36%–46%, (depending on the age group). Potential fortification vehicles, such as rice, fish/soy sauces and vegetable oil are consumed daily in significant amounts (median: 170 g/capita/day, 4 g/capita/day and 6 g/capita/day, respectively) by over 40% of the children. Vegetable oil fortification could contribute to an additional vitamin A intake of 21%–24% of VRDA recommended nutrient intake, while fortified rice could support the intakes of all the other micronutrients (14%–61% for iron, 4%–11% for zinc and 33%–49% of folate requirements). Other food vehicles, such as wheat flour, which is consumed by 16% of children, could also contribute to efforts to increase micronutrient intakes, although little impact on the prevalence of micronutrient deficiencies can be expected if used alone. The modeling suggests that fortification of vegetable oil, rice and sauces would be an effective strategy to address micronutrient gaps and deficiencies in young children.

## 1. Introduction

The critical 1000 days of human development from pregnancy until 2 years of age has received much attention from stakeholders, since inadequate nutrition during this crucial period of time is a global public health problem with long-term impact. More than one-third of deaths among children under the age of 5 years as well as other long-term consequences (stunting, reduced productivity) are due to under-nutrition during the first 1000 days [1,2]. Children under 5 years of age, and particularly those under two years of age, are often deficient in micronutrients. According to WHO, an estimated 250 million preschool children are vitamin A deficient [3]. Prevalence of anemia in children aged from 6 to 35 months can exceed 50% in countries as diverse as India, Peru and Madagascar [4]. In addition, a review estimated that zinc deficiency was responsible, globally, for 453,207 deaths per year (4.4% of childhood deaths) and 1.2% of the burden of disease among children under 5 years of age [5]. 

In Vietnam, recent studies showed that the prevalence of anemia ranges from 25% to 45% in schoolchildren [6,7]. In addition, the prevalence of zinc and selenium deficiencies were also reported to be very high (>50%) in rural Vietnamese children[8]. A nationwide survey on micronutrient status of young children carried out in 2010 in Vietnam [9] confirms these data, indicating that zinc deficiency is a severe public health problem among children aged 6–75 months, as 51.9% had plasma zinc concentrations—indicative of deficiency. While the prevalence of anemia, and deficiencies of iron and vitamin A have decreased compared to previous surveys, marginal vitamin A status is still prevalent (above 50% with plasma retinol <1.05 µmol/L), and over 25% of children (6–75 months) had a ferritin concentration <30 µg/L, indicating low iron stores. Children from 6 to 18 months of age were particularly at risk of micronutrient deficiencies [9].

While there are numerous approaches to control micronutrient deficiency using a range of interventions (fortification, supplementation, dietary diversification, other food-based approaches and public health measures), there is no single model appropriate to cover all population segments, making it important to design and implement complementary approaches to ensure the greatest penetration of fortified food products. The 6–24 month age period corresponds to the time when different foods are successively introduced to complement breast milk. The timely introduction of complementary foods and/or food supplements is generally recognized as a necessary condition to prevent malnutrition [10]. However, early introduction and poor quality of complementary foods among children less than two years of age are of substantial concern in Vietnam [11]. In addition, several studies have shown a low consumption of animal-sourced foods, such as meat or eggs, as well as other food, such as fruit [12,13]. Large-scale fortification of staple foods and condiments targeting the entire population (through mandatory or voluntary legislation) has already had some significant effects on micronutrient status among children [14,15] and could contribute to the reduction of micronutrient gaps, as children also consume family foods. For example, in Indonesia, consumption of fortified milk and noodles was associated with reduced incidence of anemia and stunting in children aged 6–59 months [16]. 

With the evidence of the impact of iron-fortified fish sauce [17,18], the Global Alliance for Improved Nutrition (GAIN) supported the development of the first national plan to launch iron fortified fish sauce to prevent iron deficiency in Vietnam. In addition, the government has issued a standard for voluntary fortification of several staples and condiments to reduce micronutrient deficiencies [19,20]. Unfortunately, the privatization of the state-run fish sauce industry in the first years of the project drastically reduced the number of participating factories and the potential impact of the project. Therefore, fish sauce fortification as a stand-alone intervention could not sufficiently reduce micronutrient deficiencies in Vietnam, and it became essential to conduct an analysis of the sauces (fish and soy sauces) and staple food (rice, wheat flour, vegetable oil) markets to examine potential additional vehicles for fortification. A market survey in Vietnam [21], commissioned in 2009 by the GAIN, showed potential for a multiple food fortification strategy due to highly concentrated industries for vegetable oil (8 producers with 95% of the market), fish sauces (13 producers with 60% of the market), flavoring powders (13 producers with 75% of the market), soy sauces (4 producers with 76% of the market) and wheat flour (8 producers with 84% of the market). In addition, rice fortification is currently getting more attention because fortification technology is now available [22,23,24,25], and new evidence is demonstrating the potential benefits of rice fortification to control micronutrient deficiencies [26,27,28,29].

By focusing on improving nutrition for mothers and children in the 1000-day window, many organizations have focused their efforts on developing new products for targeted groups. Consequently, the objective of this study was to explore, based on measured dietary intakes, whether multiple large scale food fortification of selected staple foods and condiments with multiple micronutrients in Vietnam has the potential to increase iron, folic acid, zinc and vitamin A daily intakes among children among different socioeconomic groups, age groups, and among both urban and rural populations.

The hypothesis tested was that fortification of these selected staple foods and condiments, according to existing Vietnamese food fortification regulations [19,20] would provide a significant contribution to daily micronutrient requirements of iron, zinc, vitamin A and folate in children aged between 6 and 60 months. 

## 2. Materials and Methods

### 2.1. Study Design and Sampling

The survey population of the 2009 Food Consumption Survey (FCS) consisted of 7680 households (HH), sampled from 512 clusters (104 urban and 408 rural). HH were randomly selected using a stratified 2-stage cluster sampling procedure with probability proportionate to size (PPS). A subset of HH from the FCS was selected for the 2010 micronutrient study (MNS). The sample size for the 2010 MNS was estimated on the basis of a prevalence of anemia among women of reproductive age of 50%, since in the previous national anemia survey (2005), 39.9% of the non-pregnant and 52.5% of pregnant women were anemic [30]. Therefore, a sample size of approximately 700 HH per stratum (urban/rural) was calculated to achieve an accuracy of 5.0%, with an expected design effect of 2.0. Anticipating an estimated 17% refusal or absence of women in the selected HH, 840 HH per stratum were required. Consequently, a total of 56 urban and 56 rural clusters of 15 households were selected for the 2010 MNS. Due to limited funding, sampling from each province was not possible; therefore, the National Institute of Nutrition randomly selected 19 provinces from the total of 64 provinces in Vietnam. From these 19 provinces, 56 urban clusters and 56 rural clusters were randomly chosen from those that had been included in the 2009 FCS. 

Every child (less than 5 years old) from the subset of HH was surveyed and the individual consumption data were collected (*n* = 430 children). For this paper, children less than 6 months (*n* = 15) were excluded as exclusive breastfeeding is recommended. 

### 2.2. Food Consumption Analysis

The 2009 FCS estimated the individual consumption of children using the 24-h recall method combined with controlled food weighing, similar to the procedures followed in 2000 [31]. 

The 24-h recall of amount of foods consumed by any given child was conducted by teams of dietitians or trained personnel from provincial medical centers. Dietitians interviewed the woman in charge of cooking for the child and feeding him/her. To estimate quantities of ingredients used in food preparation, various techniques were used: (1) reproduction of the quantity of food and weighing: the mother was asked to reproduce the quantity (e.g., quantity of rice cooked using the stock) or the volume of a food (e.g., volume of fish sauce using water). The enumerator weighed these quantities using a small digital balance; (2) use of a photographic food catalogue including different serving sizes of typical dishes and calibrated cooking tools (spoon of vegetable oil, spoon of sugar, *etc*.); (3) estimation according to the prices of food purchases: food prices were converted into weight using market data. 

To understand the food consumption patterns of the children, the National Institute of Nutrition (NIN) classification of food groups used for the 2000 and 2009 FCS was applied, which included a wheat flour group, a vegetable oil group, a sauces group (including fish and soy sauces) and a rice group. 

### 2.3. Socioeconomic Status

Socio-economic status was calculated from data obtained during the 2009 FCS [32], using the Demographic and Health Survey (DHS) Wealth Index to divide HH surveyed into five socio-economic quintiles: the “extreme poor” (category 1), the “poor” (category 2), the “intermediate” categories 3 and 4 and the “wealthiest” (category 5). The Wealth Index was constructed from recorded data on household assets such as tables, chairs, refrigerator, air conditioners and beds and also from housing conditions (building materials used to make the house floor; roof; main wall) and facilities (energy for cooking, electricity and latrines). Income and expenditure data were not used [33].

### 2.4. Proposed Fortification Strategy

The most prevalent micronutrient deficiencies found among children during the 2010 national-wide survey were iron, vitamin A, zinc and folate [9]. For this reason, those four micronutrients formed the basis of the current modeling exercise.

In this study, fortification levels for vegetable oil were set according to the most current national standards [19,20]. For wheat flour and rice, the latest international recommendation [34], based on an adult’s equivalent consumption of rice—over 300 g/day, and of wheat flour—below 75 g/day was adapted so as not to exceed approximately 35%–40% of the VRDA of an adult woman of 19–50 years of age. Finally, the standard for sauces was modified from 4 mg to 2.5 mg of iron as sodium iron ethylenediaminetetraacetic acid (NaFeEDTA) per 10 mL of sauce to avoid any color change or precipitation as observed in Vietnam and Thailand [35]. Table 1 presents the standard used for modeling in the present study. Sugar fortification was not included in this strategy, as the sugar consumption among adults is very low in Vietnam: 8 g/capita/day in 2000 [36]. In 2009 it was even less: approximately 6 g/capita/day [37]. 

**Table 1:** Standards used for modeling purposes of an integrated large-scale fortification strategy in Vietnam.

Food vehicle	Standards
**Wheat flour**	40 mg/kg of iron as NaFeEDTA; 50 mg/kg of zinc and 5 mg/kg of folic acid
**Rice**	40 mg/kg of iron as micronized ferrous pyrophosphate; 5 mg/kg of zinc and 0.5 mg/kg of folic acid
**Vegetable oil**	75 IU of retinyl palmitate per gram of vegetable oil
**Sauces**	2.5 mg of iron as NaFeEDTA per 10 mL of sauces

### 2.5. Statistical Analysis

In 2007, the Vietnamese government issued the VRDA [38] which recommends a higher micronutrient intake than the WHO guidelines [39]. In this analysis, the VRDA was used for assessing the relative contribution of fortification toward meeting the needs of children, as they are more tailored to the Vietnamese population. Iron bioavailability was estimated to be low (5%) for diets rich in cereals [39]. A low absorption of zinc was also assumed, considering the diet in Vietnam is rich in phytate [39]. 

Data entry, management and analysis, including quality checks, were performed using SPSS software version 19™ (IBM Corporation, New York, USA). To calculate daily energy, macro- and micronutrient intakes the database was linked to the Vietnamese Food Composition Database [40]. Descriptive statistics were used to examine all variables. Consumption distribution curves were asymmetric according to the test of Kolmogorov-Smirnov and therefore only non-parametric tests were performed. Median and interquartile range values of the *per daily capita* consumption of children are presented and disaggregated by socioeconomic status (quintile), age group, sex and residence (urban/rural). Food consumption (continuous variables) was compared by groups by using the non-parametric Kruskal-Wallis test (more than two groups) or Mann-Whitney U-test (two groups) for independent variables. 

The median consumptions of nutrients through fortifiable food (such as rice, wheat flour, vegetable oil and flavoring powder) by age group were calculated by multiplying the level of fortificant (including the losses during processing and storage: 5% for iron and zinc, 30% for vitamin A and 50% for folic acid [39]) by the amount of food consumed.

### 2.6. Ethical Issues

The Scientific Committees of the National Institute of Nutrition (Hanoi, Vietnam) and of the Ministry of Health (Hanoi, Vietnam) reviewed and approved the study protocol.

All households were informed verbally and in writing about the aims and procedures of the study, and written informed consent was obtained from the person in charge before enrollment.

## 3. Results

Food consumption patterns of 415 children (51.1% boys and 48.9% girls) were analyzed, of which 46.3% were urban and 53.7% were rural. Among these children, 24.0% were living in the “extreme poor” HH (category 1), 16.9% in the “poor” HH (category 2), 14.5% in intermediate low HH (category 3), 18.1% in intermediate high (category 4), and 26.5% in the “wealthiest” HH (category 5). 

The children’s median energy intake from their diet was 871.2 Kcal/day, with a significant difference between age groups (from 597.8 Kcal/day for children aged between 6 and 11.9 months, to 957.9 Kcal/day for children aged between 36 and 59.9 months). No significant differences were observed when correlated for sex or residence (see Table 2) for all the intakes. Among age groups, significantly higher quantities of protein and carbohydrates were consumed by older children *versus* infants (for protein: 35.6 g/day *vs*. 22.7 g/day; *p* < 0.001, and for carbohydrates: 159.1 g/day *vs*. 69.6 g/day; *p* < 0.001). Differences can be observed also among socioeconomic groups, as less protein and lipids were consumed by the poorest *versus* the wealthiest groups (for protein: 23.4 g/day *vs*. 36.3 g/day; *p* < 0.001, and for lipids: 9.4 g/day *vs*. 16.4 g/day; *p* < 0.001).

The median per capita per day consumption levels of iron, zinc, vitamin A (as retinol activity equivalent), vitamin B_1_ and B_2_ from the diet were respectively: 4.8 mg, 4.3 mg, 182.8 µg, 0.5 mg and 0.3 mg (Table 2). Again, no significant differences were observed in median iron, zinc, vitamin A, vitamin B_1_ and B_2_ when correlated against sex and residence, while significant differences were noted for median iron, vitamin A, zinc, vitamin B_1_ and B_2_ between the two groups: age and socioeconomic level. 

**Figure 1:**
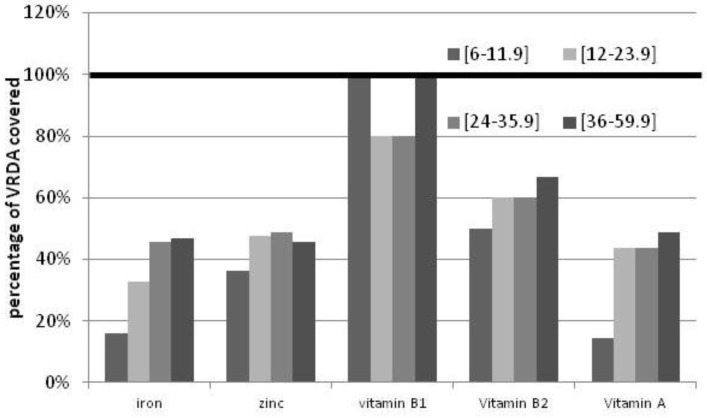
Percentage of children achieving the VRDA for retinol, zinc, iron, vitamin B1 and vitamin B2 by age group.

As shown in Figure 1, using the VRDA and depending on the age group, the median intake of children covers from 16.1% to 46.8% of the iron requirement, from 36.1% to 48.8% of the zinc requirement, from 14.4% to 48.9% of the vitamin A requirement, from 80.0% to 100.0% of the vitamin B_1_ requirement and from 50% to 66.7% of the vitamin B_2_ requirement.

**Table 2:** Macro- and micronutrient intake (in g/capita/day) by rural and urban residence, sex, age group and socioeconomic group (median and 25th, 75th percentile) ^1,2,3,4^.

	*n*	Energy (Kcal)	Protein (g)	carbohydrates (g)	Lipid (g)	Iron (mg)	Zinc (mg)	Vitamin A (µg retinol activity equivalent)	Vitamin B1 (mg)	Vitamin B2 (mg)
**Total**	415	871.2 (617.9; 1199.3)	33.1 (22.0; 45.0)	140.7 (101.3; 199.9)	15.4 (8.2; 23.8)	4.8 (3.1; 7.4)	4.3 (3.0; 6.2)	182.8 (40.0; 387.9)	0.5 (0.3; 0.7)	0.3 (0.2; 0.5)
**Sex**										
Boy	212	884.2 (642.5; 1229.3)	34.7 (22.7; 46.0)	144.8 (103.5; 217.7)	16.3 (8.5; 23.8)	4.9 (3.2; 7.3)	4.5 (3.1; 6.5)	177.8 (40.3; 383.8)	0.5 (0.3; 0.7)	0.3 (0.2; 0.5)
Girl	203	830.3 (601.0; 1126.4)	32.3 (21.5; 44.8)	131.7 (97.3; 185.8)	14.3 (7.3; 23.8)	4.7 (2.9; 7.4)	4.2 (2.9; 6.1)	190.7 (37.1; 397.3)	0.5 (0.3; 0.7)	0.4 (0.2; 0.6)
*p * ^2^		*p* = 0.090	*p* = 0.373	*p* = 0.127	*p* = 0.179	*p* = 0.592	*p* = 0.113	*p* = 0.837	*p* = 0.392	*p* = 0.476
**Age groups (month)**										
(6–11.9)	48	597.8 (327.4; 871.0)	22.7 (11.3; 36.0)	69.6 (37.8; 110.6)	11.5 (3.8; 21.6)	3.0 (1.6; 4.1)	3.0 (1.7; 6.1)	57.7 (4.5; 224.1)	0.3 (0.1; 0.7)	0.2 (0.1; 0.4)
(12–23.9)	97	760.7 (555.5; 1143.2)	27.2 (17.8; 40.9)	111.8 (82.3; 202.5)	14.9 (7.8; 23.4)	3.8 (2.4; 5.5)	4.0 (2.4; 5.7)	174.6 (49.4; 284.9)	0.4 (0.2; 0.8)	0.3 (0.1; 0.5)
(24–35.9)	94	883.2 (647.0; 1173.8)	34.7 (21.6; 45.5)	148.4 (117.0; 189.8)	14.4 (8.2; 25.7)	5.3 (3.6; 8.3)	4.1 (2.9; 6.2)	174.4 (39.5; 430.7)	0.4 (0.3; 0.7)	0.3 (0.2; 0.6)
(36–59.9)	176	957.9 (732.5; 1269.2)	35.6 (25.6; 48.5)	159.1 (118.6; 225.4)	16.3 (9.8; 23.7)	5.9 (4.2; 8.6)	4.7 (3.6; 7.0)	220.2 (78.1; 448.6)	0.6 (0.3; 0.8)	0.4 (0.2; 0.6)
*p * ^2^		*p* < 0.001	*p* < 0.001	*p* < 0.001	*p* = 0.159	*p* < 0.001	*p* < 0.001	*p* < 0.001	*p* < 0.01	*p *< 0.001
**Category socioeconomic**										
Extremely poor	100	733.8 (578.0; 1039.7)	23.4 (17.9; 35.5)	139.3 (103.5; 192.6)	9.4 (3.0; 19.7)	3.9 (2.7; 5.8)	3.5 (2.2; 4.9)	121.8 (3.0; 273.5)	0.3 (0.2; 0.6)	0.2 (0.1; 0.4)
Poor	70	990.0 (737.8; 1225.0)	36.6 (25.5; 47.0)	161.1 (119.0; 250.8)	16.3 (11.6; 22.6)	5.4 (3.7; 7.2)	4.7 (3.5; 6.6)	199.9 (73.9; 449.4)	0.6 (0.3; 0.8)	0.4 (0.2; 0.6)
Intermediate low	60	883.2 (608.5; 1176.4)	34.8 (22.1; 48.0)	136.3 (101.4; 188.4)	16.2 (8.6; 26.8)	4.9 (2.7; 8.0)	4.6 (2.9; 6.4)	153.7 (32.9; 350.4)	0.5 (0.3; 0.8)	0.4 (0.1; 0.6)
Intermediate high	75	833.1 (551.7; 1247.3)	34.8 (23.5; 47.4)	132.7 (80.4; 173.3)	18.5 (8.9; 28.7)	5.0 (2.9; 8.2)	4.5 (2.9; 6.9)	259.9 (103.2; 430.7)	0.6 (0.3; 0.8)	0.4 (0.2; 0.6)
Wealthy	110	900.7 (686.3; 1248.0)	36.3 (25.4; 48.6)	134.9 (99.2; 214.0)	16.4 (10.0; 26.1)	5.1 (3.8; 8.1)	4.8 (3.3; 6.4)	186.7 (59.9; 430.3)	0.5 (0.3; 0.7)	0.4 (0.2; 0.6)
*p * ^2^		*p* < 0.01	*p* < 0.001	*p* = 0.148	*p* < 0.001	*p* < 0.01	*p* < 0.01	*p* < 0.001	*p* < 0.01	*p* < 0.001
**Areas**										
Rural	223	916.7 (613.7; 1199.3)	32.8 (21.5; 44.1)	151.4 (99.9; 201.0)	16.1 (8.5; 23.6)	5.0 (3.2; 7.4)	4.3 (3.1; 6.4)	177.5(32.4; 372.0)	0.5 (0.3; 0.7)	0.3 (0.2; 0.5)
Urban	192	804.2 (625.5; 1209.0)	34.4 (22.4; 47.1)	132.9 (102.0; 197.4)	14.7 (8.0; 24.7)	4.7 (3.1; 7.3)	4.4 (2.9; 6.2)	183.3 (53.0; 401.0)	0.5 (0.3; 0.8)	0.3 (0.2; 0.6)
*p * ^2^		*p* = 0.363	*p* = 0.396	*p* = 0.354	*p* = 0.768	*p* = 0.988	*p* = 0.868	*p* = 0.308	*p* = 0.671	*p* = 0.271

^1^ median (25th percentile; 75th percentile); ^2^ test of Kruskal-Wallis or Mann-Whitney U-test on the median; ^3^ no data exists for folate in the Vietnamese food table; ^4^ not including intakes from breastfeeding.

Rice (Ri) was consumed daily by almost all children. Fish and soy sauces (S) were consumed by half of the children, while wheat flour (WF) was consumed daily by less than one sixth of children and vegetable oil (VO) by more than a third of children (Table 3). 

**Table 3:** Percentage of Vietnamese children consuming selected food, by socioeconomic status (quintile), sex, age groups and residence in the previous 24 h.

	*n*	Rice	Wheat flour	Vegetable oil	Sauces
**Socioeconomic status**					
Extremely poor	100	100.0%	19.0%	18.0%	47.0%
Poor	70	100.0%	15.7%	34.3%	54.3%
Intermediate low	60	96.7%	20.0%	41.7%	50.0%
Intermediate high	75	97.3%	12.0%	45.3%	50.7%
Wealthy	110	96.4%	15.5%	52.7%	52.7%
*p* ^1^		*p* < 0.05	ns	*p* < 0.001	ns
Sex					
Boys	212	100.0%	16.5%	41.5%	52.4%
Girls	203	96.6%	16.3%	35.0%	49.3%
*p* ^1^		*p* < 0.05	ns	ns	ns
**Age groups (month)**					
(6–11.9)	48	91.7%	4.2%	45.8%	62.5%
(12–23.9)	97	100.0%	17.5%	28.9%	47.4%
(24–35.9)	94	96.8%	9.6%	38.3%	42.6%
(36–59.9)	176	100.0%	22.7%	41.5%	54.0%
*p* ^1^		*p *< 0.05	*p* < 0.01	ns	ns
**Areas**					
Rural	223	100.0%	18.8%	33.6%	51.1%
Urban	192	96.9%	13.5%	43.8%	50.5%
*p* ^1^		*p* < 0.05	ns	*p* < 0.05	ns
**Total population**	415	98.6%	16.4%	38.3%	50.8%

Note: ^1^ Chi square test; ns: non-significant.

The proportion of children consuming vegetable oil and rice significantly differed by socioeconomic status, as the proportion of poor children consuming VO is less as compared to the wealthiest groups of children while it is the opposite for rice consumption. The proportion of children consuming sauces and wheat flour did not differ among socioeconomic groups. Analyses of different combinations of food vehicles showed that from the age of 6 months, 45.8% to 61.0% of the children were consuming on a daily basis VO-Ri while between 20.8% and 30.2% of the children were consuming VO-S on a daily basis. The other combinations (WF-VO; Ri-WF-VO-S; WF-VO-S) were consumed daily by less than 10% of the children (from 1.1% to 9.4%). Vitamin A fortification of vegetable oil has been shown to be the most cost-effective, simple-to-implement strategy [41] and therefore VO was the choice in the different combination to increase the vitamin A intake.

Among consumers, the median consumption of rice, wheat flour, vegetable oil and sauces for children was 170 g/day, 43 g/day, 6 g/day and 4 g/day respectively (Table 4). The median consumption of rice and sauces varied significantly among different age groups while vegetable oil varied among residence (rural/urban). 

**Table 4:** Median consumption (25th percentile; 75th percentile) for consumers of selected foods by socioeconomic status, sex, age groups and residence (in g/capita/day) ^1^.

	*n*	Rice	*n*	Wheat flour	*n*	Vegetable oil	*n*	Sauces
Total	408	170.0 (110.0; 250.0)	68	43.0 (14.3; 76.0)	159	6.0 (3.0; 10.0)	211	4.0 (2.0; 9.0)
Sex								
Boy	212	180.0 (120.0; 263.0)	35	50.0 (15.0; 80.0)	88	6.0 (3.0; 10.0)	111	4.0 (2.0; 8.0)
Girl	196	157.0 (104.0; 240.0)	33	35.0 (12.5; 75.5)	71	6.0 (3.0; 9.0)	100	4.5 (2.5; 9.0)
*p * ^2^		*p* = 0.150		*p* = 0.507		*p* = 0.583		*p* = 0.926
**Category socioeconomic**								
Extremely poor	100	165.0 (111.8; 243.0)	19	50.0 (14.0; 76.0)	18	5.0 (3.0; 9.8)	47	4.0 (2.0; 7.0)
Poor	70	204.4 (126.0; 295.0)	11	40.0 (12.0; 110.0)	24	6.5 (4.0; 11.5)	38	5.0 (2.0; 10.5)
Intermediate low	58	170.0 (117.8; 254.0)	12	45.0 (15.6; 92.8)	25	5.0 (3.0; 9.0)	30	6.0 (2.0; 8.3)
Intermediate high	73	150.0 (100.0; 219.0)	9	35.0 (12.0; 51.5)	34	6.0 (4.0; 9.3)	38	5.0 (3.0; 9.0)
Wealthy	106	164.0 (108.8; 247.8)	17	50.0 (18.5; 67.5)	58	5.5 (3.0; 10.6)	58	4.0 (2.0; 9.5)
*p * ^2^		*p* = 0.168		*p* = 0.813		*p* = 0.661		*p* = 0.683
**Age groups (month)**								
(6–11.9)	44	70.0 (47.5; 120.0)	2	6.0 (6.0; 6.0)	22	5.5 (2.0; 15.0)	30	2.5 (2.0; 7.5)
(12–23.9)	97	125.0 (93.5; 250.0)	17	40.0 (16.0; 75.5)	28	6.0 (3.3; 13.2)	46	4.0 (2.0; 6.0)
(24–35.9)	91	185.0 (124.0; 230.0)	9	50.0 (23.5; 88.8)	36	5.5 (4.0; 9.8)	40	3.5 (1.6; 8.8)
(36–59.9)	176	195.0 (136.5; 284.5)	40	47.0 (12.8; 84.5)	73	6.0 (3.0; 9.0)	95	5.0 (3.0; 10.0)
*p * ^2^		*p* < 0.001		*p* = 0.150		*p* = 0.864		*p < 0.05*
**Areas**								
Rural	223	175.0 (105.0; 270.0)	42	47.5 (19.5; 81.9)	75	6.0 (3.0; 15.0)	114	5.0 (2.0; 11.3)
Urban	186	167.5 (114.8; 247.8)	26	35.0 (12.0; 71.3)	84	5.0 (3.0; 9.0)	97	4.0 (2.0; 7.0)
*p * ^2^		*p* = 0.883		*p* = 0.167		*p* < 0.05		*p* = 0.196

^1^ median (25th percentile; 75th percentile); ^2^ test of Kruskal-Wallis or Mann-Whitney U-test on the median.

The oldest children consumed significantly more rice (*p* < 0.001) and sauces (*p* < 0.05) (195 g/capita/day and 5 g/capita/day respectively) than most of the other age groups. For vegetable oil, rural children consumed significantly more than those living in urban areas (6.0 g *versus* 5.0 g, *p* < 0.05). No significant difference was shown for the median consumption of rice, vegetable oil, wheat flour and sauces among socioeconomic groups (Table 4), even though the percentage of children consuming vegetable oil significantly tended (*p* < 0.01) to increase with increasing socioeconomic status (Table 3).

**Figure 2:**
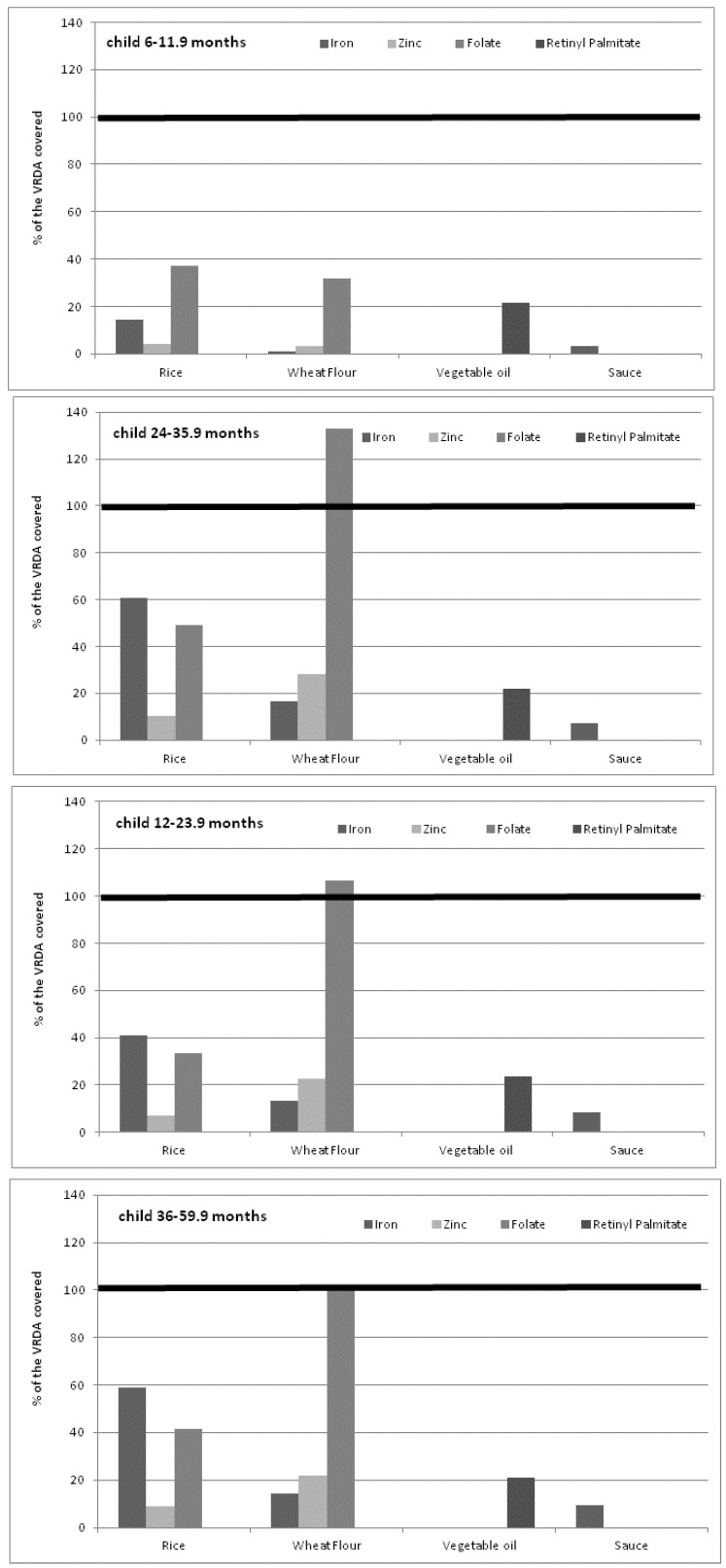
Percentage of VRDA for retinol, zinc, iron and folate contributed by potential consumed fortified food vehicles.

Estimates of the amounts of iron, folic acid, zinc and vitamin A provided by individual fortified food vehicles (Ri, WF, VO and S) and the contribution to the daily VRDA for children depending on their age were calculated in Table 5 and Figure 2. 

**Table 5:** Estimated daily contribution from individual fortified foods (using median amounts and proposed fortification levels consumed daily by children) ^1,2^.

	Nutrient contribution per child from individual food fortification							
Age groups	Fortified wheat flour			Fortified sauces	Fortified vegetable oil	Fortified rice		
	Iron (mg/day)	Zinc (mg/day)	Folate (µg/day)	Iron (mg/day)	Retinyl palmitate (µg RAE/day)	Iron (mg/day)	Zinc (mg/day)	Folate (µg/day)
**(6–11.9) **	0.2 (0.2–0.2)	0.3 (0.3–0.3)	26 (26–26)	0.6 (0.5–1.8)	86.6 (31.5–236.3)	2.7 (1.8–4.6)	0.3 (0.2–0.6)	30 (20–51)
**(12** **–23.9) **	1.5 (0.6–2.9)	1.9 (0.8–3.6)	170 (68–321)	1.0 (0.5–1.4)	94.5 (52.0–207.9)	4.8 (3.6–9.5)	0.6 (0.4–1.2)	53 (40–106)
**(24** **–35.9) **	1.9 (0.9–3.4)	2.4 (1.1–4.2)	213 (100–377)	0.8 (0.4–2.1)	86.6 (63.0–154.4)	7.0 (4.7–8.7)	0.9 (0.6–1.1)	79 (53–98)
**(36** **–59.9) **	1.8 (0.5–3.2)	2.2 (0.6–4.0)	200 (54–359)	1.2 (0.7–2.4)	94.5 (47.3–141.8)	7.4 (5.2–10.8)	0.9 (0.6–1.4)	83 (58–121)

^1^ Values are medians (25th and 75th percentiles); ^2^ assuming 5% losses for iron, 30% losses for retinyl palmitate and 5% losses for zinc.

Fortified VO and S, consumed respectively by 38% and 51% of the children, could provide from 21.0% to 23.6% of the VRDA for vitamin A and from 3.2% to 9.4% of the VRDA for iron, depending on age group (Figure 2). Fortified Ri, consumed by 98.6% of the population and significantly different among age groups (Table 4), could provide a VRDA of between 14.3% and 60.6% of iron, 4.0% to 10.5% of zinc and between 33.2% and 49.1% of folate. Moreover, fortified WF, among those who consumed it, would provide a VRDA intake per child of 1.2% to 16.4% of iron, 3.4% to 28.3% of zinc and 31.9% to 132.8% of folate.

Combining all potential fortifiable foods in Vietnam (Ri-VO-S-WF), a combination consumed by less than 10% of the children older than 6 months shows that this multiple food fortification strategy, depending on age group, would ensure a VRDA intake per child of 18.7% to 84.2% of iron (0.8 mg to 3.0 mg iron through NaFeEDTA and 2.7 mg to 7.4 mg iron through micronized ferrous pyrophosphate), 69.1% to 181.9% for folate (56 µg to 292 µg), 7.4% to 38.8% for zinc (0.6 mg to 3.3 mg) and 21.0% to 23.6% for vitamin A (86.6 to 94.5 µg of retinyl equivalent). 

In Vietnam, more than one third of children over 6 months of age consume both rice and vegetable oil daily. If rice and vegetable oil were fortified, depending on their age group, these children would be provided a range between 2.7 mg and 7.4 mg of micronized ferrous pyrophosphate, 30 µg to 83 µg of folate and 0.3 mg to 0.9 mg of zinc from rice and 86.8 to 94.4 µg of retinyl equivalent from fortified vegetable oil.

## 4. Discussion

Based on analysis of the subsample of the 2009 FCS, the daily diet does not provide sufficient amounts of micronutrients, such as iron, zinc and vitamin A. Large-scale mass fortification of staple foods and/or condiments would reach a significant segment of children (16.4% to 98.6% depending on the food vehicle) and could increase the micronutrient intake of the children consuming these foods. For example, in Vietnam, the consumption of rice is widespread, with more than 90% of children over 6 months of age consuming it daily. The figures per capita/day consumption broken down by age are: 70 g for children aged 6 to 11.9 months, to 195 g for children aged 36 to 59.9 months. Thus, fortification of rice could play an important role in preventing micronutrient deficiencies. 

A review of infant and young child feeding practices in Vietnam was recently published [42], and highlighted that the feeding practices of infants and young children have not changed significantly over the last decade. Phuong *et al*. emphasized the need to continue development of more affordable and nutritious complementary foods for the poor in Vietnam. However, these types of interventions require changes in child feeding practices, which require behavior change interventions. A French NGO (GRET) has faced this challenge; after several years of intervention, only 13% of the targeted families were purchasing the fortified product at least once per month [43]. Our paper emphasizes the importance of (among other interventions) large-scale fortification of staples and condiments that are already used by mothers, in order to increase micronutrient intakes among children. A large advantage is that this strategy does not require behavior change.

As shown by the modeling exercise, based on expected fortification levels and median consumption rates, two food vehicles (rice and sauces) are widely consumed daily by over 50% of children, regardless of area of residence (urban *vs*. rural) and socioeconomic status. Vegetable oil, consumed by more than a third of the children, is also a good vehicle to increase the intake of retinyl palmitate. Therefore, their fortification should be one of the priorities for stakeholders. This would contribute to filling the gap of micronutrient intake as measured through the 24-h recall (Figure 1). Unfortunately, the Vietnamese food composition tables do not provide reliable information on other nutrients (such as vitamin B_12_ and folate) to generate a more complete overview of gaps in micronutrient intake in vulnerable groups. 

### 4.1. Vegetable Oil Fortification: A Solution for Additional Vitamin A Intake and Possibly Vitamin D Intake

The 2010 MNS [9] showed that 11.8% of children under two years of age and 11.9% of the children between 2 and 5 years of age were VAD deficient. More than half of children had a marginal or deficient vitamin A status. Vitamin A is a vital nutrient for the functioning of the immune system as well as for the healthy growth and development of children [44]. This high prevalence of marginal vitamin A status would suggest that efforts to prevent vitamin A deficiency have to be maintained and even strengthened, especially for children under the age of two. A diverse diet rich in animal food sources that contain preformed vitamin A can be sufficient to meet the daily requirement for vitamin A [45]. However, with the inflation of food prices in developing countries such as Vietnam [46], studies show that populations prioritize calorie-rich but nutritionally poor foods [47,48]. As highlighted in this study, the current diet, according to age groups, provides 14.4% to 48.9% of the retinol requirement.

Eliminating or preventing vitamin A deficiency through vitamin A supplementation has also been the focus of universal efforts and needs to continue. However, when reviewing the sustainability of the increase in serum retinol concentration following a periodic provision of high doses of vitamin A, the literature reveals mixed results. Two studies in the Philippines and India show that the effect of high dose vitamin A capsules lasts between 2 and 3 months with no impact after 6 months. [49,50]. This would suggest that other complementary types of interventions, such as food fortification, are necessary, as supplementation alone appears to be insufficient for maintaining nutritional reserves beyond 3 months. Fortification of vegetable oil with vitamin A is considered a cost-effective and easily implementable strategy [41]. Retinyl palmitate is the most commonly used vitamin A fortificant, and has been used successfully to fortify sugar, monosodium glutamate, and wheat flour, in addition to cooking oil [51,52,53]. If vegetable oil were to be fortified, it would contribute between 21% and 23.6% of the VRDA for children because of its widespread consumption.

In addition, studies among Asian children and African American teenagers also suggested that low dietary intakes of calcium result in increased catabolism of vitamin D and the development of vitamin D deficiency and rickets [54]. Unfortunately, no published data exist on the deficiency of vitamin D among children in Vietnam. According to the 2010 MNS (personal communication[55]), more than 50% of children were vitamin D deficient (below 50 nmol/L [56]), and more than 30% with “insufficient” levels (circulating 25(OH)D between 51 and 74 nmol/L [56]). This preliminary data indicates that prevention of vitamin D deficiency in children is a public health priority that needs to be addressed through appropriate interventions. Vegetable oil may be a suitable vehicle for fortification with vitamin D, as well as retinyl palmitate, however this proposal would require further dietary modelling within the Vietnamese context. 

### 4.2. Rice and Sauces Fortification to Increase Zinc, Iron and Folate Intakes

The latest 2010 MNS study highlighted that over 25% of children had low iron stores [9]. Recent reviews suggest that iron deficiencies in early life have persistent negative effects later in life [57,58]. The fortification of rice has proven to be effective in improving iron status for children (between 6 and 13 years old) in India when the fortified rice was provided, containing at least 17 mg to 19 mg iron through micronized ferrous pyrophosphate [29,59]. In Vietnam, where the diet provides approximately 4.8 mg of iron, if children age 6 to 59.9 months consumed fortified rice with micronized ferrous phosphate and sauces with NaFeEDTA, those products could provide, depending on the age groups, ranges of 2.7 mg to 7.4 mg, and 0.6 mg to 1.2 mg respectively. This additional intake of iron is not negligible, as NaFeEDTA is known to enhance the absorption of both the intrinsic food iron [60] and iron used in other fortified foods, and thus may have a beneficial effect on overall iron absorption from the diet.

According to the analysis, the Vietnamese diet provides over 40% of VRDA of zinc to children; in addition, almost 52% of children [9] are considered zinc deficient, based on the actual serum zinc cut-off. Fortified rice would be able to provide zinc, and no adverse effects have been shown from zinc fortification [61]. The United States Food and Nutrition Board of the Institute of Medicine estimated the upper limit (UL) for zinc to be 7 mg of zinc for children 1–3 years and 12 mg for children 4–8 years [39]. The combination of the current diet and the fortification of rice would provide a maximum of between 6.7 and 7.3 mg of zinc for children between 1 and 3 years, and 8.4 mg for children between 4 and 6 years of age (considering a median consumption of rice and zinc consumption through the diet at the 75th percentile level, the worst scenario). Therefore, research is needed to estimate the optimal quantity of zinc to be added through fortification of staples and condiments. 

For folate, the Vietnamese food composition tables do not provide sufficient information. Children consuming rice at the 75th percentile level would consume an additional 51 μg to 121 μg of folate which is far from the UL estimated for children 1–3 years (300 μg) and for children 4–8 years (400 μg) [39].

Rice fortification is currently attracting more attention and stakeholders, together with international agencies, are trying to develop models to create a sustainable market in a rice producing country. In addition, new technologies introducing fortified rice through village rice millers are now available and could be a solution in Vietnam [62]. 

### 4.3. Wheat Flour Fortification to Increase Zinc, Iron and Folate Intakes: A Possibility but Not a Priority

Wheat flour fortification is more logistically feasible than rice fortification because there are only 20 flourmills in Vietnam using imported wheat flour. In addition, wheat flour is often fortified around the world. Wheat flour fortification could provide multiple micronutrients at the same time, such as iron, zinc and folic acid. Unfortunately, the downside of flour fortification is that it will not reach most of the Vietnamese population, regardless of socioeconomic status, sex and residence. The reason, according to the survey, is that between 75% and 80% of children do not consume wheat flour. So, if the percentages of wheat flour consumers among the extreme poor (19.0%) and all children (16.4%), are taken into account, little impact on rates of deficiencies through this vehicle would be expected.

Even if wheat flour were to be fortified in Vietnam, it is essential to evaluate the risk for reaching the UL for a population that, in the future, might be eating rice, wheat flour and sauces. For example, if we consider children aged between 24 and 35.9 months who consume rice, wheat flour and sauce at the 75th percentile level, fortification of all these food vehicles could provide 14.2 mg of iron, 5.3 mg of zinc and 475 μg of folate. It may also be taken into account that zinc bioavailability from rice may be higher compared to that of wheat flour, because of the lower inhibitory effect of phytate on zinc (higher molar ratio before absorption is inhibited). The combination of the current diet and the fortification of food vehicles would provide nutrients of 22.5 mg *i.e*., below the 40 mg UL for iron for children 1–3 years, but unfortunately, could be above the UL for zinc (11.5 mg *vs*. a UL of 7 mg). Zinc is a nutrient that is likely to exceed the UL if fortification of wheat flour were to be implemented, and further modeling is required in this regard.

### 4.4. Limitations of the Study

The population analyzed for this article is a subsample of the FCS sample that was also used for the MNS [9]. However, this subsample has not biased the results, as similar findings from the dietary intake of energy, macronutrients and micronutrients were found among the 7980 households surveyed [63].

The 24-h recall data provided single day consumption and therefore the percentage of children consuming may be underestimated, because those that did not consume on the day before the interview may consume another day that week. In addition, the average intake among consumers might be overestimated because it is assumed that the intake on the previous day represents average daily intake, whereas consumption may not take place every day. Finally, the 24-h recall does not capture seasonal variation, such as the consumption of different fruits rich in various micronutrients, or nutrients provided from breast milk.

## 5. Conclusions

When developing strategies for young children to prevent micronutrient deficiencies, it is important to estimate the potential contribution from a large-scale food fortification program to micronutrient intakes. The results of this study show that fortification of rice, sauces and vegetable oil that are consumed daily by a large proportion of Vietnamese children would be an effective strategy to reduce micronutrient deficiencies, whereas fortification of other vehicles, such as wheat flour, would have less impact on improving the micronutrient status of vulnerable populations. 
